# Electro dialysis reversal (EDR) performance for reject brine treatment of reverse osmosis desalination system

**DOI:** 10.1371/journal.pone.0273240

**Published:** 2022-08-24

**Authors:** Hossein Ataei Far, Amir Hessam Hassani, Lobat Taghavi, Mojtaba Fazeli, Abdollah Rashidi Mehrabadi

**Affiliations:** 1 Department of Environmental Science, Faculty of Natural Resources and Environment, Science and Research Branch, Islamic Azad University, Tehran, Iran; 2 Department of Water and Wastewater Engineering, Faculty of Civil, Water and Environmental Engineering, Shahid Beheshti University, Tehran, Iran; South China University of Technology, CHINA

## Abstract

In this study, the performance of bench-scale EDR was evaluated using the samples taken from the 1st and the 2nd stage RO from the Brackish Water Reverse Osmosis (BWRO) plant in Eshtehard, Iran. The measurements indicated that original TDS of the aquifer brackish water was equal to 3,229–3,664 mg/L, whereas TDS of the 1^st^ stage RO brine was between 5,500 and 7,700 mg/L, that TDS of the 2^nd^ stage RO brine was in the range of 9,500–10,600 mg/L. A batch bench-scale EDR system of 12 l/h was used with a direct electric current at three different scenarios. In the first, the brine was fed at 20°C (as a reference regulated point). In the second, temperature (14, 20, 26.5°C), and in the third, voltage were changed (6, 12, 18, 24 V) to investigate their influences on performance of the EDR process, while the other operational parameters (feed flow rate, recovery ratio, quality of feed brine)were kept constant. Based on the data analysis using the ANOVA and DUNCAN tests for the second and third scenarios, it was observed that the optimum TDS removal efficiency of the EDR process can be at temperature of 26.5°C and voltage of 18 V. On the other hand, the successful performance of the bench-scale EDR in reducing the 29,000 mg/L TDS and the 45,000 μmhos/cm EC of the 2^nd^ stage brine to 1,716 mg/L (TDS) and 2,640 μmhos/cm (EC) (at 26.5°C and 24V) could be considered as the main achievement of this research. Overall, the hybrid process RO-EDR-RO can be considered as the best technical, environmental and economical scenario for the development of Eshtehard Desalination Plant phase 2 at full scale.

## 1. Introduction

According to UN World Water Development Reports, water scarcity has become a major issue for the world’s population, and the situation is expected to become more critical in the next two decades [1–4]. Desalination has been proposed as one possible solution to alleviate pressures on freshwater resources [5–8]. The desalination systems are divided into three major groups: condensation, membrane, and other processes (freezing, hybrid, etc.).

The share of electrodialysis in the global desalination process has been stated as 2% and the share of electrodialysis reversal as 1% [9]. In the research plan for compiling the roadmap of new water desalination technologies in Iran, the Water Research Institute of the Ministry of Energy has mentioned the share of electrodialysis as equal to 2%.

There are 15,906 operational desalination plants around the world producing around 95 million m3/day of desalinated water for human use (48% in the MENA Region). A major challenge associated with desalination technologies is the production of a typically hypersaline concentrate discharge (around 142 million m3/day), that requires disposal, which is both costly and associated with negative environmental impacts [9, 10]. The Zero-liquid discharge (ZLD) approach has been recently considered as a significant solution to protect the environment and to achieve sustainability [11].

In this section, the plan’s background and the records of electrodialysis projects’ operation, as well as research projects of relevance, are explained. The REvivED water plans (21 research projects) were initiated with the aims of drinking water supply through state-of-the-art technologies in brackish/seawater desalination, at the low-cost recovery of water. In this context, Campione et al. conducted their research on coupling the electrodialysis desalination process with photovoltaic and wind energy systems [12].

In addition to presenting the scientific basics, they analyzed the parameters effective on the performance of electrodialysis [13]. Karimi and Ghassemi studied the impacts of parameters on the performance of a bench-scale EDR that used brackish waer of 1700 μS/cm in electrical conductivity [14]. Xu et al. manufactured a bench- and pilot-scale EDR of reclaimed water to determine the impact of operational parameters on effluent quality to be used for irrigation. The membrane’s surface in the pilot-scale EDR was observed without scaling or fouling through the three-month continuous testing period [15]. Benneker et al. studied the impact of temperature gradient on ED and reverse electrodialysis (RED) as the significant regime. The results indicated that as the temperature of the feed water increased from 20 to 40°C, the power required by the ED process to desalinate seawater without changing the membrane selectivity and the level of desalination, decreased down to 15% [16]. Valero et al. presented the theory and application of ED, including design, operation, and maintenance, and two case studies [17]. In addition, Valero et al. studied the three-year operation of the EDR plant at Abrera Drinking Water Treatment Plant in Spain. The results of this study underlined the need for periodical inspection and maintenance of the electrodialysis’ membranes during their full-scale operation [18].

Rehman and Ahdab studied the application of Monovalent Selective ED process. The results indicated that the produced water containing low monovalent ions and appropriate concentration of divalent ions can be used as fertilizer, and could entail a savings of up to 4,995 $/ha per year. This process can be readily applied to treat the 6,000 brackish groundwater resources in the United States [19].

This research investigated the performance of bench- scale EDR in treating the real brine from the 1st and 2nd stage RO of the Eshtehard BWRO desalination plant, with a TDS concentration of up to 29,400 mg/l.

Moreover, by applying the results of this research and following the technical analysis of the construction and operation costs, the different alternatives for treating the brine in the plan to upgrade the Eshtehard desalination plant were evaluated, and the best technical and environmental options for the industrial-scale full-scale plant was proposed.

The novelty of this research lies in applicability of its results as an intermediate treatment process in BWRO desalination plants which focuses on the removal of divalent ions (with tendency for scaling), and in its potential for private sector participation in development of the phase 2 of Eshtehard desalination plant, in the light of technical, environmental and economic evaluations.

## 2. Materials and methods

**Fig 1:**
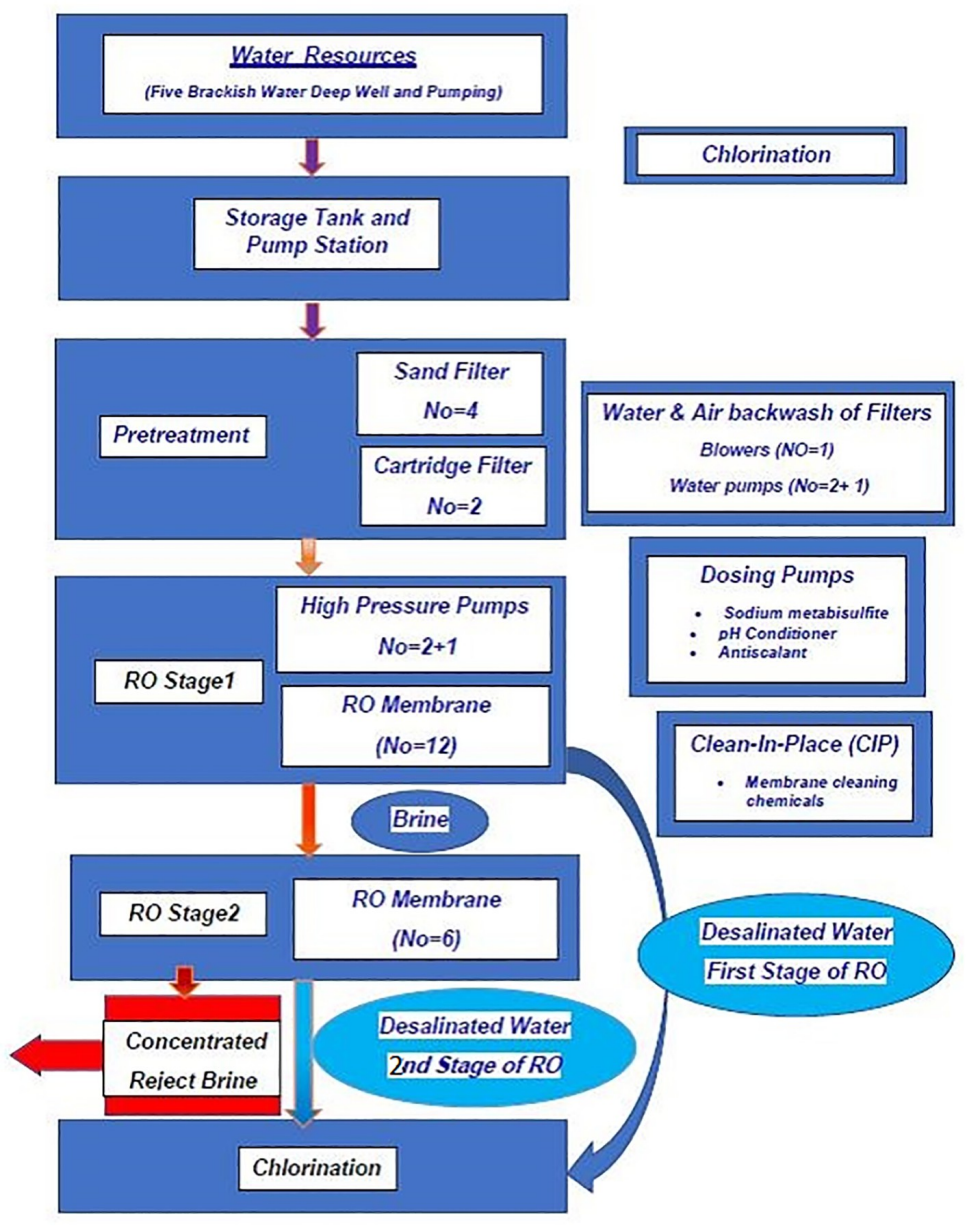
Pre-treatment and the 2 stage RO processes of the BWRO desalination plant (samples taken from the 1^st^ and 2^nd^ stage brine of the studied plant).

This research was built on an empirical study to evaluate the performance of bench-scale EDR using the actual brine (with samples taken from the brine of a BWRO desalination plant in Eshtehard, a city of Alborz Province (Iran) at different scenarios. No synthetic samples were used in the bench-scale EDR to realize a practical application of the results in treating the system’s brine. The desalination plant (the site of sampling) was completed and operated since 2016 (brackish water is withdrawn from an aquifer) as a two-stage RO system that includes pretreatment (Sand filtration, filter cartridge, disinfection), chemicals, and antiscalants injection and the RO membrane cleaning (CIP) as shown in Fig 1. The brine from the 1^st^ stage was transferred to the 2^nd^ stage membrane for a greater recovery and production of water.

### 2.1. The specifications of the bench-scale EDR

**Fig 2:**
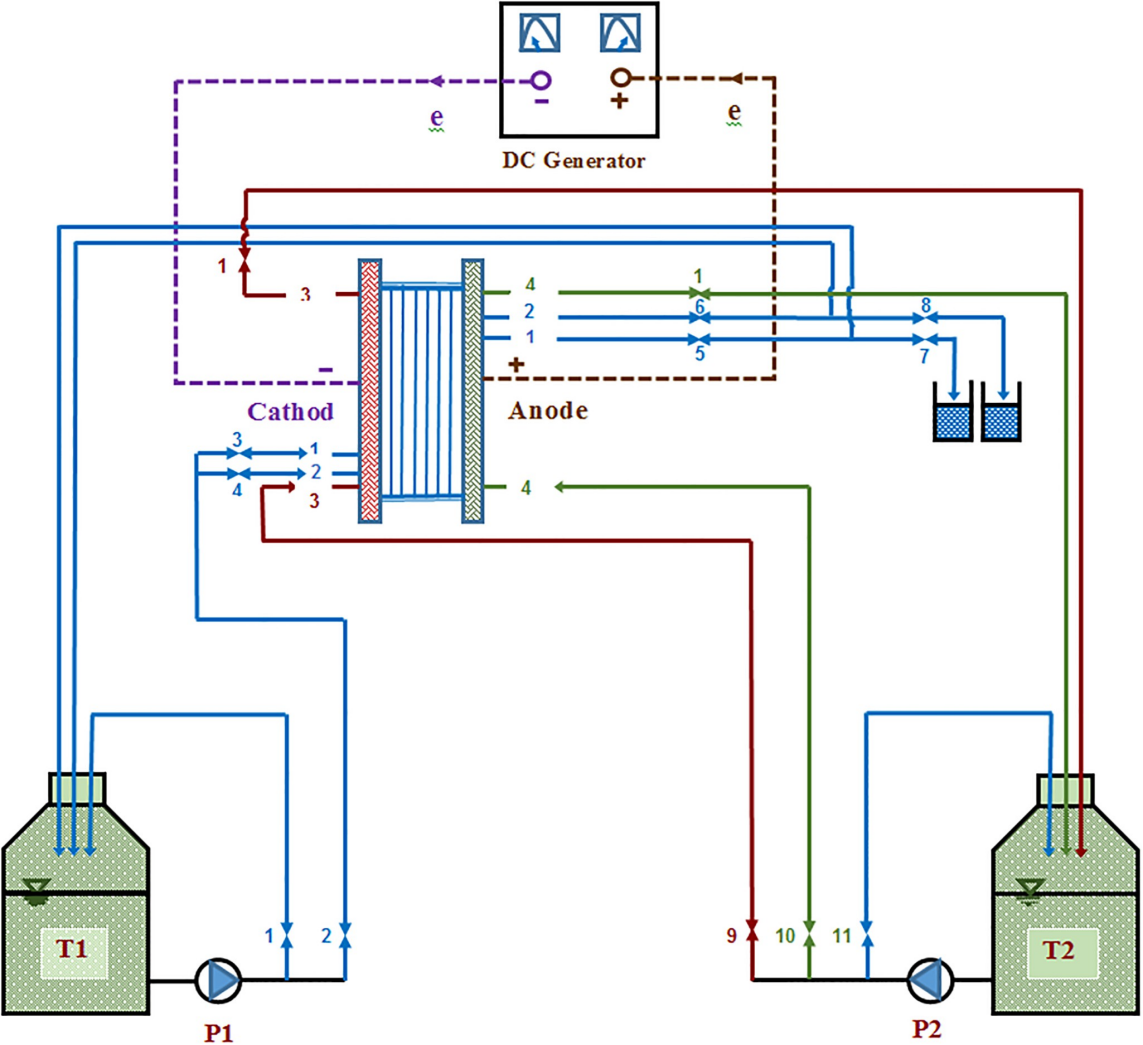
The components of the EDR bench -scale used in this research. T1: concentrated solution tank; T2: electrolyte solution tank; P: electro-pump; DC generator: Direct current generator; Cathode: the cathode pole of electrodialysis; Anode: the anode pole of electrodialysis. (The design and construction of the bench -scale electrodialysis were realized with the collaboration of Mr. Amirreza Arashi).

A bench-scale EDR system equipped with Cationic Exchange Membranes (CEM) and Anionic Exchange Membranes (AEM) installed in alternating pattern with spacers between electrodes, was assembled in a plexiglass vessel of 12 l/h in capacity. Spacers were incorporated with the gaskets to prevent the contact of cation-exchange membranes with anion-exchange membranes and the solutions mixing. Attaching the electrodes to the rectifier made it possible to apply direct current at different voltages to the bench-scale electrodialysis apparatus. The bench-scale was made from Plexiglas and the valves were of corrosion-resistant polymers. The CEM and AEM were of type 10 manufactured by Fujifilm Company, while the electrodes were made of titanium with platinum coating and the lab’s rectifier was produced by Sanjesh Co. (a domestic firm). The anionic membrane had a perm selectivity of 95%, water permeability of 6.5 mL/m^2^.h.bar, and a resistance of 1.7 Ω.cm^2^ (low resistance), while the cationic membrane’s permselectivity was equal to 99% (high perm selectivity). The bench-scale was then set up in preparation to test the brine. The direct electrical current was supplied from a source (the AC-DC transformer with voltage regulation). Fig 2 shows schematic diagram of the EDR bench-scale’s components used in this research.

In the first scenario, the brine was fed at 20°C (as a reference regulated point). In the second, temperature (14, 20, 26.5°C), and in the third, voltage were changed (6, 12, 18, 24 V) to investigate their influences on the performance of the EDR process, while the other operational parameters (feed flow rate, recovery ratio, feed quality) were kept constant.

The independent variables of the study included TDS, hardness, alkalinity, pH, and other quality parameters of the feed and the study-dependent parameters included the quality and the quantity of the produced water and the concentrate. Since the changes in the voltage and the amperage of the electrodialysis throughout the operation were an indication of the increased electrical resistance of the device, the calibrated voltage and the amperage of the rectifiers were checked regularly and the electrodialysis polarity was reversed as needed. Depending on the experimental parameters, a time from 20 to 40 min was considered before the data gathering to obtain a steady-state process.

### 2.2. Operating conditions

There are more parameters to be studied. However, based on the preliminary experiments, the ones that are more effective on the EDR operation, i.e. voltage and temperature which could be adjusted, were selected.

The bench-scale system was operated at different temperatures in the range of 14–26.5°C in one scenario and different voltages in the range of 6–24 V while the other parameters were kept constant. The samples were taken from the feed brine, the desalinated water, and the concentrate of the device. They were tested according to the Standard Methods for the Examination of Water and Wastewater 2017, 23rd Ed.

The tests were conducted with calibrated lab equipment and according to methods stipulated by the Code of the standards. The pH, Turbidity, Electrical conductivity, Sodium & Potassium, and Sulfate & Nitrate contents were determined using laboratory equipment respectively Metrohm 744 pH Meter, Turbidimeter HACH 2100AN, Conductometer Metrohm 912, Flame Photo meter (Jenway PFP7), and Spectrophotometer (HACH DR6000). The Calibration Standard Certified Reference Material was used for calibration in the process of the tests. For example, to calibrate the test for the total hardness of water or brine we used the CaCO3 1000mg/L Calibration Standard, which has the chemical code of CRM-008-500.

## 3. Results and discussion

The measurements of the raw water and the brine samples of the Eshtehard desalination systems showed that the system’s feed brine contained a TDS of 3,229 to 3,664 mg/L; the 1^st^ stage RO brine had a TDS of between 5,500 and 7,700 mg/L, while the TDS of the brine from 2^nd^ stage RO was in the range of 9,500–10,600 mg/L. In the first scenario, the bench-scale tests were conducted in September 2020 for a feed brine temperature of 20°C as a calibration point. In the second scenario, the impact of temperature increase, and in the third scenario, the impact of voltage change on the electrodialysis’ performance were studied. In this scenario, tests were carried out on the feed brine at 20°C containing TDS and salinity concentrations of 9,000 and 10,600 mg/L with an applied voltage of 24 V on the electrodialysis. The TDS of the desalinated water was in the range of 500–1200 mg/L. The results obtained from these tests in this scenario are as follows:

- A high drop in pH value to 3.5 indicated the acidity and corrosiveness of the desalinated water, which required the addition of lime or other materials to increase its alkalinity.
- The high efficiency of nitrate removal and the production of water with a nitrate content of less than 5 mg/L, confirmed the high efficiency of electrodialysis for this process.

In the second scenario according to Table 1, the impacts of temperature variation on the performance of the electrodialysis were studied at temperatures of 14 and 26.5°C with the electrodialysis operating at a voltage rating of 24 V.

**Table 1:** The TDS removal percentage of the EDR at different feed temperatures. (Feed flow rate = 12 L/h, water recovery ratio for TDS 6764 mg/L = 75% and for TDS 9434 mg/L = 52% and TDS 29341 mg/L = 23% the applied voltage = 24 V).

Feed	% of TDS removal
T 14°C & TDS 9,434 mg/L	81%
T 20°C & TDS 10,631 mg/L	89%
T 26.5°C & TDS 29,341 mg/L	94%

In the second scenario, by comparing the tests on the feed brine at 14 and 26.5°C, having a TDS of 9,434 and 29,341 mg/L, respectively, the following results were obtained:

- The great drop in pH value of the produced water to 3.5 underlined its acidity and corrosiveness, which had to be adjusted by increasing the alkalinity (by adding lime or other materials) to ensure its stability.
- At 14°C, the electrodialysis’ efficiency in removing hardness, calcium and magnesium from a feed brine containing a TDS concentration equal to 9,400 mg/L was 84%, and its efficiency in removing chloride, sulfate and nitrate, was 86, 80 and 86%, respectively.
- At 26.5°C, the electrodialysis’ efficiency in removing calcium and magnesium from a feed brine containing a TDS concentration equal to 29,300 mg/L was 95%, and its efficiency in removing chloride, sulfate and nitrate, was 94, 96 and 96.2%, respectively.

In the third scenario, the tests were conducted by changing the voltage (6, 12, 18 and 24 volts) and keeping constant the values of other parameters effective on the electrodialysis’ performance. The results of the tests indicated that the highest efficiency was achieved at 18V according to Table 2, during which, the TDS and EC were reduced by 93.71% and the removal ratios of hardness, Calcium, Sodium and Potassium were in order 98, 98.03, 98.1, 98.6 and 90%.

**Table 2:** TDS removal percentage in EDR at different voltages. (Feed flow rate = 12 L/h, water recovery ratio for TDS 6764 mg/L = 75% and feed temperature = 14°C).

Feed Voltage (V)	TDS removal (%) (Feed TDS = 6764 mg/L)	Hardness removal (%) (Feed TDS = 6764 mg/L)
^6^	59.0	69.5
^12^	83.0	93.4
^18^	93.0	98.0
^24^	77.8	86.0

**Fig 3:**
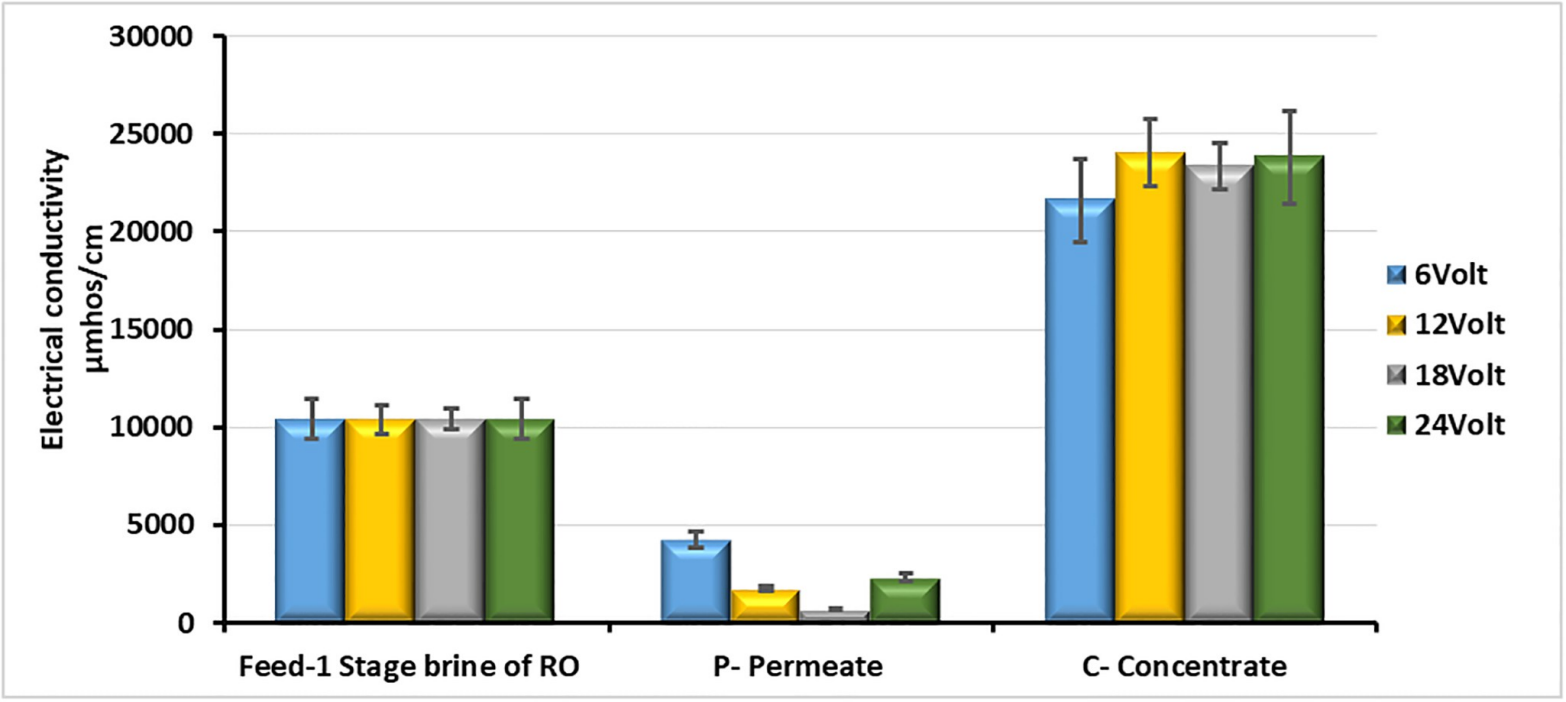
Comparison of the EC of the feed brine, the desalinated water, and the concentrated brine at 4 different voltages (feed flow rate = 12 L/h, water recovery ratio for TDS 6764 mg/L = 75% and feed temperature = 14°C).

The results of these tests indicated that the applied voltage was an important factor in enhancing the performance of electrodialysis, to the extent that for brine containing a TDS concentration of 6,764 mg/L, the electrodialysis operating at 18 V and 14°C, achieved a removal efficiency of 94%, as shown in Fig 3.

**Fig 4:**
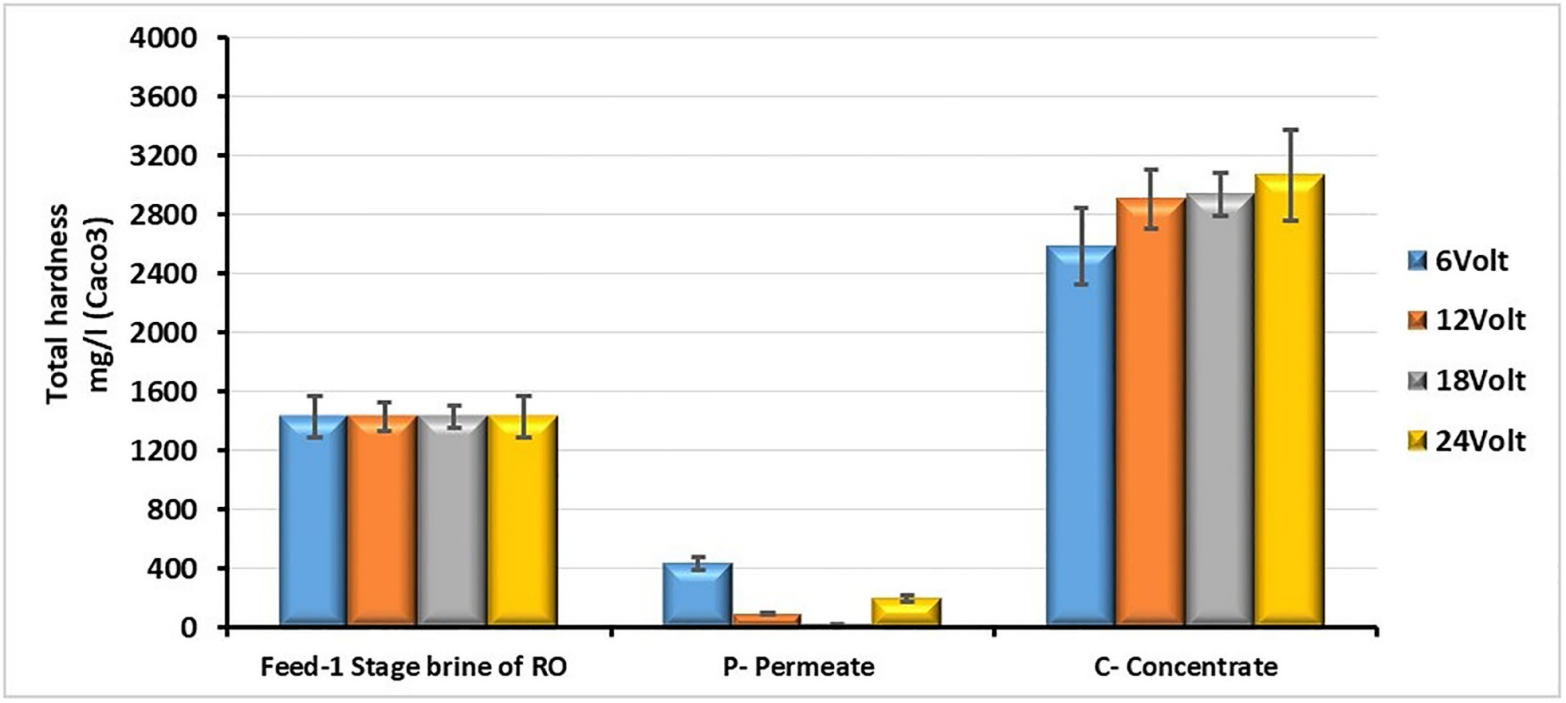
Comparison of the hardness of the feed brine, the desalinated water and the concentrated brine at different voltages (feed flow rate = 12 L/h, water recovery ratio for TDS 6764 mg/L = 75% and feed temperature = 14°C).

It can be observed that the removal of hardness by electrodialysis reaches a ratio of 98% at 18 V, and it can be emphasized that electrodialysis is among the best methods for hardness removal and softening of water, as shown in Fig 4. The trend of monovalent and divalent ions removal at all voltages, with the highest ion removal efficiency achieved at 18 V, is shown in Table 3.

**Table 3:** The percentage of cation and anion removal in EDR at different voltages. (Feed flow rate = 12 L/h, water recovery ratio for TDS 6764 mg/L = 75% and the feed temperature = 14°C).

Feed	Ca^2+^	Mg^2+^	Na^+^	K^+^	SO_4_^2-^
Voltage (V)	removal (%)	removal (%)	removal (%)	removal (%)	removal (%)
6	72/4	52/7	62/8	50	63/6
12	93/4	93/5	93/1	50	92
18	98	98/1	98/6	90	96/4
24	86/7	86/7	87/9	84	79/1

The measurements show that the bench-scale EDR has the TDS and hardness removal efficiency of 93 and 98%, respectively, the Calcium, Magnesium and Sodium removal efficiency of 98% and the sulfate and Potassium removal efficiency of 96 and 90%, respectively at 18V. Thus, the EDR can be considered as an intermediary treatment of the 1^st^ stage RO of the Eshtehard BWRO desalination plant for the efficient removal of divalent ions with a high scaling tendency.

### 3.1. Statistical analysis

Table 4 shows the statistical analysis of the trend of TDS removal from the brine at different temperatures and voltages (scenarios 2 and 3). The Student’s T mean comparison test was applied to compare the TDS removal by the bench scale EDR at different feed temperatures and applied voltages. The effect of temperature in reducing the TDS with a p-value of equal to 0.07534 with 90% confidence level and the effect of voltage with a p-value equal to 0.02726 with 95% confidence level were evaluated as acceptable. Moreover, the results of DUNCAN Test indicated that the feed temperature of 26.5°C had the greatest effect on TDS removal, while 18 V was considered as the most effective voltage.

**Table 4:** The statistical analysis of the trend of TDS removal from the brine at different temperatures and voltages (the second and the third scenarios).

Brine temperature (°C)	Changes in % of TDS removal (6 V)	Changes in % of TDS removal (18 V)
14.0	48.10±10.20	81.5±1.5
20.0	59.12±7.75	93.71±4.6
26.5	63.25±4.5	96.5±2.24

### 3.2. Effect of brine and electrodialysis feed samples’ quality on deposit

The Langelier index was calculated throughout the tests to study the scaling or non-scaling tendency of the feed brine. The overall results of quality analyses along with the studies of Langelier and Ryznar indexes of the feed water, the permeate and the brine from the 1st and 2nd stage RO of the Eshtehard desalination plant, as well as the results of these indexes for different feed qualities of the bench-scale EDR and even the Stiff-Davis index of the feed with a TDS of 29,341 mg/L indicated the feed being of the non-scaling quality. The concentrate in all samples was non-scaling, except for the one with a TDS concentration of 50,362 mg/L, which showed a scaling tendency according to the Langelier Index (2.1) and the Ryznar Index (4.4). The stability index of feed water, the permeate and the brine of the Eshtehard desalination plant and the feed brine, the concentrate, and the produced water of the EDR are shown in Table 5, while the relevant calculations are presented in the Supporting Information File.

**Table 5:** The stability index of the feed brine and the permeate of the Eshtehard desalination plant and in the bench-scale EDR.

Feed Characteristics	TDS (mg/L)	Langelier Saturation Index (LSI)	Ryznar Saturation Index (RSI)	Quality of LSI	Quality on basis of RSI
Feed Quality					
RO	3229	-0.5	8.8	No Deposition	No Deposition
EDR	6764	0.4	7.5	Low deposition	No Deposition
EDR	9431	-1	8.7	No Deposition	No Deposition
EDR	29341	- 0.5	8.8	No Deposition	No Deposition
Brine and Concentrated Quality					
RO	9579	0.08	7.3	Low deposition	No Deposition
RO	5407	-0.06	7.9	No Deposition	No Deposition
EDR	15172	-7.63	17.4	No Deposition	No Deposition
EDR	15508	-0.44	7.9	No Deposition	No Deposition
EDR	50562	2.1	4.4	Deposition	Deposition
Water Desalinated Quality					
RO	29	-2.4	12.	No Deposition	No Deposition
RO	279	-1.99	11.5	No Deposition	No Deposition
EDR	425	-8.2	19.4	No Deposition	No Deposition

The measurements showed that due to their low pH value (6.5), the 2nd stage RO brine of the Eshtehard desalination plant and the EDR concentrate does not tend to scale. Therefore, the EDR can be used as an intermediary treatment unit.

### 3.3. Quality of the electrodialysis desalinated water and its comparison with standards

**Fig 5:**
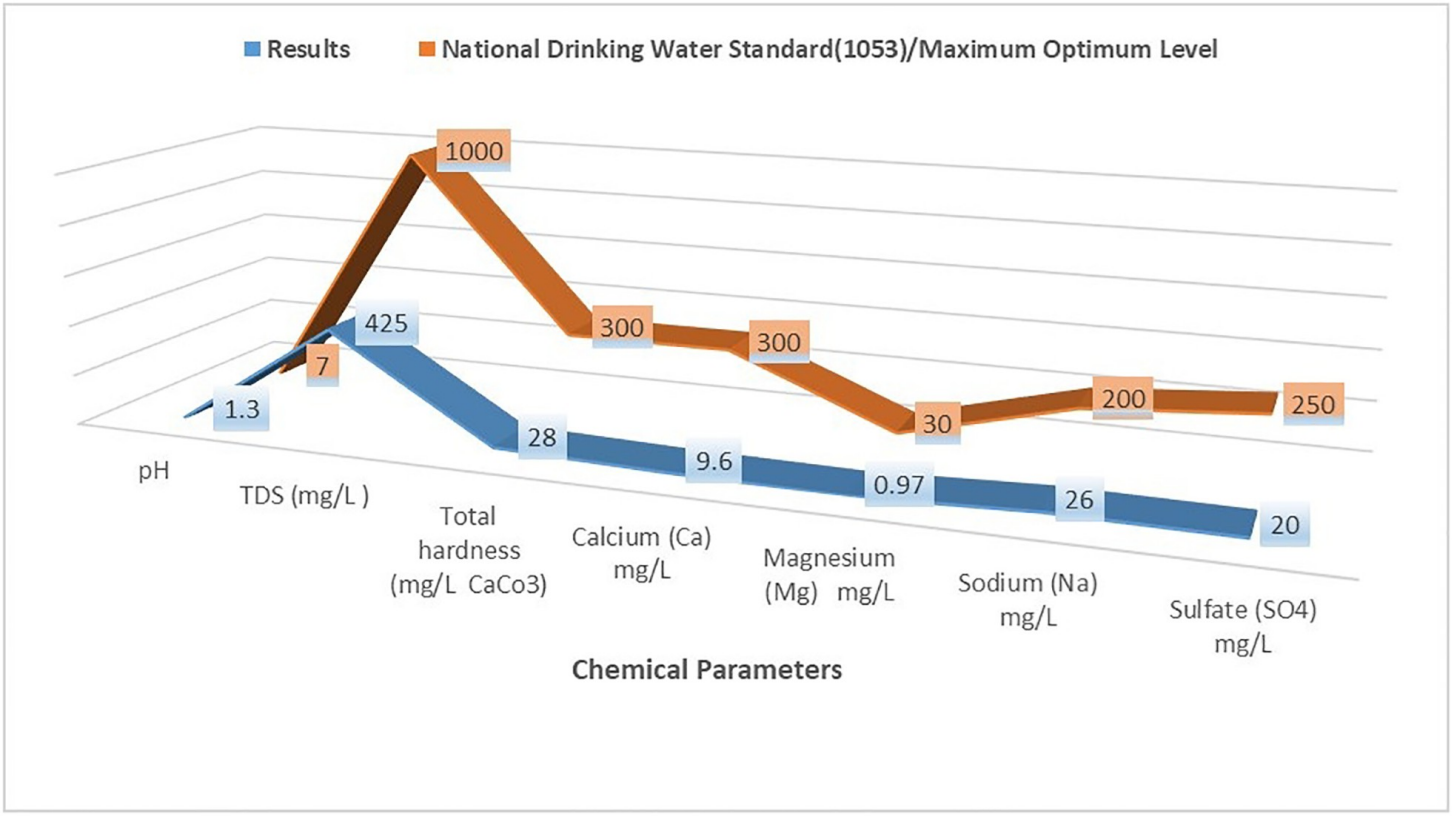
Comparing the quality of the bench-scale electrodialysis’ desalinated water to the national standards for drinking (Standard 1053).

Suppose the quality of the bench-scale electrodialysis desalinated water is to be compared with the Iranian National Standards (1053). In that case, it becomes clear that it is within the desirable range of the national standards (Drinking Water Standard -Physical & Chemical Properties of Water -2012). Still, it needs to be adjusted given its low pH value. Moreover, due to the low mineral contents, especially the cations, the quality of relevant parameters in the permeate must be increased to the limits of the determined values. Fig 5 shows the quality of the bench-scale electrodialysis’ permeate compared to the National Standards for Drinking Water.

Suppose the quality of the desalinated water from the bench-scale electrodialysis is compared with the physical and chemical criteria of Drinking Water Regulations Produced by Desalination Plant (regulation 20704—published by the Ministry of Health of Iran). In that case, it becomes clear that despite its TDS concentration being within the limits of desirable quality (desirability of TDS equal to 200 mg/L), its hardness and its divalent cations are less than the regulation values (desirable of hardness and Calcium and Magnesium concentrations equal to 200 mg/L CaCO3 and 50 and 20 mg/L, respectively). Therefore must be adjusted and improved to the levels specified.

Moreover, the test results indicated that electrodialysis can remove the brine’s nitrate at 99% efficiency (the reduction of Nitrate concentration from 52.25 mg/L in the feed brine to 0.38 mg/L in the desalinated water). Even when the feed brine TDS concentration reached 29,000 mg/L, the efficiency of the bench-scale electrodialysis is still 93% (the reduction of Nitrate concentration from 33.25 mg/L in the feed brine to 2.29 mg/L in the desalinated water). Therefore, electrodialysis can serve the dual purpose of desalination and Nitrate removal.

### 3.4. Factors limitations and potentialities the electrodialysis’s efficiency

The results of the studies on the bench-scale electrolysis show the minimum limiting current to be equal to 0.6 A for 20 V, which promotes polarization thereafter when the applied voltage and reduction of the system’s efficiency. To reduce the intensive polarization in the electrodialysis, solutions such as reducing the intensity of the electrical current, improving the hydraulic conditions and redesigning cells and the types of spacers have been proposed. The brine from the 1^st^ and 2^nd^ RO stages in the mentioned systems had good clarity, and, and the values of turbidity being less than 1 NTU (max. 5 NTU), SDI less than 3, TOC less than 0.5 mg/L. The feed water of the RO membranes of the desalination plant showed it to be less than 3 and the saturation indexes denoted a no scaling tendency, indicating good quality of the EDR feed.

Since the biological parameters were removed and neutralized by the processes of pretreatment (Sand filtration and filter cartridge) and chlorination before entering the membrane units, the biofouling reached its minimum level.

Moreover, by pretreatment and adjusting the feed’s pH to improve its quality, controlling the voltage and current and reversing the polarity as needed, regular cleaning of the membranes and reducing the amount of recovery at high TDS values of the brine, and continuous motoring of EC and any changes in the produced water and the concentrate and other parameters. The fouling was kept to a minimum throughout the bench-scale EDR’s operation. Since the desalinated water is acid and has a low pH value, (in the range of 3.8 to 5.5), necessary measures must be taken to adjust its pH and prevent its corrosives (by injecting it with lime water or passing it over a lime bed) to ensure its quality for drinking purposes. Moreover, to compensate for the deficiency of minerals, especially Calcium and Magnesium in the desalinated water, it is necessary to either blend or apply another alternative solution according to the local conditions.

As there was a possibility of scaling from deposition of Calcium, Magnesium, and Sulfate, on the bench-scale EDR, measures such as polarity reversal in addition to using the reversal valve every 20 min, regular rinsing of the membranes with deionized water, and regular cleaning of the bench-scale EDR with a weak acid acetic (overhaul) were implemented.

In this research, the design and the operation of EDR were undertaken successfully. Kabay et al. concluded that the change of voltage from 5 to 10 V has a more significant effect on the removal of monovalent ions than the divalent ions, especially when both are present in the solution [20]. As far as the ion removal by electrodialysis was concerned, it was found that at the applied voltage of 12 V, the removal rate of divalent ions was greater than monovalent ions [21].

The most desirable properties of the ion exchange membranes are high selectivity, low electrical resistance, appropriate mechanical resistance, a stable form, and appropriate chemical stability. An issue raised in the operation of the membrane systems is the use of antiscalants. Their Deposition may increase biofouling on the membrane surface [22]. Based on the research of fouling conducted by Tofighy & Mohammadi et al. the surface waters have a greater tendency for biofouling than the groundwater resources [23]. Rezaei et al. studied to identify the fouling agents on 6 SWRO modules (EC = 55 000 μ s/cm, turbidity = 11 NTU), and found that due to the high SDI of feed water, colloidal materials and the malfunction of the pretreatment process (chlorination and neutralization, filter cartridges, coagulation and filtration), fouling was occurred on membranes. The accumulation of metallic compounds on membranes was due to the fact that no antiscalants were used in the SWRO system [24]. Numerous investigations have been conducted by Melnik et al. [25], Popov et al. [26], Andrianov et al. [27], and other researchers on environmentally friendly and biodegradable “green” antiscalants materials. Other advantages of EDR operating at a lower pressure and process were quiet operation (the elimination of the high head pressure pumps), lower maintenance requirements and ease of operation with periodic measurements (feed, brine, and desalinated water flow rates and control the voltage and amperage and the other parameters). Moreover, by pretreatment and adjusting the feed’s pH to improve its quality, controlling the voltage and current and reversing the polarity as needed, regular cleaning of the membranes and reducing the amount of recovery at high TDS values of the brine, and continuous motoring of EC and any changes in the produced water and the concentrate And other parameters.

The main limiting factor in the operation of bench- scale electrolysis in this research was the polarization.

### 3.5. Comparison of different technical, economic and environmental alternatives on full scale

Since the current capacity of Eshtehard desalination plant is 5000m^3^/d and an equivalent capacity is considered for phase 2 of its development, three different proposed scenarios of:

- RO–RO
- RO–EDR–EDR
- RO–EDR- RO

were evaluated from technical, environmental and economic aspects to select the best scenario. As in Iran, the private sector participates in the construction and operation of desalination systems in the frame of BOO contracts, the financial models of this approach were reviewed in the above-mentioned scenarios, as shown in Tables 6 and 7.

**Table 6:** The criteria of financial model for phase 2 of the Eshtehard hybrid brackish water desalination plan.

Model’s assumptions	Year
Construction period of the plant, including civil and construction works	2
Operation period of the desalination plant	16
Service life of the equipment for direct calculation of depreciation	16
Service life of RO membrane	5
Service life of EDR membrane	8

**Table 7:** The CAPEX & OPEX and price of electrical energy consumption of each scenario (1USD = 300,000 Iranian Rials).

Scenarios	Desalination modules	Capital cost to produce one m3 of water ($/ m3)	Operation costs of each one m3 of water* (Cent/m3)	Specific Energy consumption index per each m3* (KW-h/m3)	The price of 100 KW-h of energy at peak consumption (Cent)
The development phase using the 2-stage RO	1st Stage of RO	300	6	0.8	0.3
	2nd Stage of RO	400	12	2.5	
The development phase using the 2-stage RO + the EDR module	RO	300	6	0.8	0.3
	1st Stage of EDR	400	11	1.2	
	2nd Stage of EDR	600	13	2	
The development phase using RO and EDR + RO	RO	300	6	0.8	0.3
	1st Stage of EDR	400	11	1.2	
	2nd Stage of RO	400	12	2.5

1- The costs of each scenario were calculated based on the information collected from the relevant references and finalized according to the prices quoted by the desalination investment companies for the year 2022.

Furthermore, the three scenarios were evaluated according to the following three cash flow indexes:

2- The Net Present Value (NPV) is the difference between the present value of income and the present value of the costs at the intended rate of return (15%). If the difference is positive, the project has an economical justification, i.e. the present income value is larger than the present value of the costs.
3- The Internal Rate of Return (IRR) is the decremental rate, according to which the NPV of the investment plan becomes zero. In other words, it is the decremental rate according to which, the NPV of the income becomes equal to the costs. If the IRR is larger than the rate of interest (e.g. a bank interest rate of 18%), then the plan is feasible and profitable.
4- The Payback Period (PP) is the period during which, the revenues compensate for the capital investment. For analysis, it is better for the payback period to be short, since there is less assurance about a distant future than a near one. Thus, the shorter payback period, the lower the investment risk. The criteria for the growth of operating costs and water tariffs are presented in Table 8.

**Table 8:** The other criteria and the growth rates of operation costs and water tariffs.

Assumptions	Rate of financing (minimum expected rate of return)	Tax	Growth of operational and energy costs	Growth of water tariffs
**Scenario 1** ^**st**^	**15%**	**25%**	**18%**	**18%**
**Scenario 2** ^**nd**^	**15%**	**25%**	**18%**	**18%**
**Scenario 3** ^**rd**^	**15%**	**25%**	**18%**	**18%**

Based on the financial models presented in the Supporting Information File, the results of the three scenarios evaluated according to the above mentioned criteria are presented in Table 9.

**Table 9:** The results of the financial models of the three proposed scenarios.

Results	NPV	IRR	PP
Scenario 1^st^	1,818,791	0.29	5
Scenario 2^nd^	2,119,748	0.27	7
Scenario 3^rd^	2,191,886	0.28	5

The results of the financial models of the three scenarios can be concluded as follows:

Since, at the minimum expected rate (15%), the net present values (NPV) of costs and revenues during a 16-year operation will be positive, the plan’s implementation at 1/3 USD (100,000 Iranian Rials) per cubic meter of water will have an economic justification in all the three proposed scenarios. Since their internal rates of return (IRR) are larger than the expected rate of return (15%), the plan at all the three scenarios will be profitable. As the three scenarios have a positive NPV, the one with a higher NPV has a greater justification. Moreover, the plan with a greater IRR yields a greater economic return than others. Furthermore, the plan with a shorter payback period, has a priority over the others. Here, the NPV of the third scenario and the IRR of the first scenario are larger than the others, while the payback periods of both scenarios are equal. Therefore, the choice is the more efficient one. Since, the two NPVs and IRRs are based on the assumption that the generated revenues can be reinvested in a new project at a specified rate of return, but that this reinvestment can only be possible by considering the rates of financing about IRR. Therefore, NPV is a more reliable criterion than IRR for comparing and grading the investment project. Accordingly, since the third scenario has a higher NPV, it has a higher priority over the others.

As the third scenario offers the best financial aspect, it was also investigated for its environmental and technical advantages, with the following results demonstrating its priority over the others:

The practical application of EDR as an intermediary treatment method for desalination, and in particular as a measure to reduce the divalent ions with a tendency for scaling in the reject brine, offers many important environmental and technical advantages. These include the reduction of fouling and scaling in the 2nd stage RO membranes; reduced amount of antiscalants and increased water and flux recovery, increased service life of the RO membrane, extending the intervals of membrane cleaning, and considering the plan as a measure to counter the effects of drought and to minimize the volume of reject brine from the desalination plant.

In conclusion, the RO-EDR-RO scenario with a water recovery rate of 95% has greater technical, environmental and economic advantages and is proposed as the selected scenario for investment in phase 2 development of Eshtehard BWRO desalination plant.

## 4. Conclusions

The results of this research project indicated that the electrodialysis reversal EDR, with an easy operation, can be used to treat the brine from desalination plants. Increasing the temperature and the applied voltage can enhance the performance of the electrodialysis process.

Based on the statistical analysis, and by applying the ANOVA test at a confidence level of 95% for the second scenario, the supposition of a difference in the averages of TDS removal at the three mentioned temperatures was accepted. By applying the DUNCAN test, it was determined that the greatest most significant impact occurred at the temperature 26.5°C. Through the application of ANOVA test in the third scenario to determine the impacts of 4 voltage levels, the supposition of the difference in the average of these four levels was accepted with a confidence level of 95%. Moreover, through the application of DUNCAN test, it was established that the optimum level of TDS removal occurred at 18 V.

Thus, in the second scenario, the bench-scale electrodialysis was able to reduce the TDS in the feed brine from 29,300 to 1,700 mg/L in the desalinated water at a temperature of 26.5°C, while in the third scenario, the concentration of brine TDS up to 6,764 mg/L was turned to drinking water containing 425 mg/L of TDS with a cation and anion removal efficiency of 95% at 18 V and at 13°C.

In general conclusion, it can be claimed that the best performance of the bench-scale electrodialysis in reducing or removing TDS occurred at 26.5°C and 18 V. Moreover, to ensure the optimum conditions, in addition to undertaking due diligence in the design and construction of the electrodialysis system, it was necessary to continuously monitor the important parameters affecting its performance.

According to the results, the electrodialysis reversal process offers advantages such as high desalination efficiency; efficient removal of the brine’s hardness, cations and anions; as well as the ease of operation at different alternatives to address the issues of fouling & scaling; while its limitation lies in the possibility of polarization (because of the intensity of the limit flow).

Moreover, to achieve a green desalination system, special attention must be paid to the treatment and disposal of the rejected brines of the BWRO desalination plants. In this regard, based on the studies undertaken, the desalination of brackish water and the brine reject from the RO process could be recycled with the feed stream. In conclusion, the RO-EDR-RO scenario with a water recovery rate of 95% has greater technical, environmental and economic advantages and is proposed as the selected scenario for investment in phase 2 development of Eshtehard BWRO desalination plant.

## Supporting information

S1 Table Average of chemical parameters in EDR bench scale. (DOCX)

S2 Table Langelier’s and ryznar’s indices. (XLS)

S3 Table The financial model for phase 2 of the eshtehard hybrid brackish water desalination plan. (XLSX)

S1 Text Calculation S&DSI for feed brine in EDR bench-scale. (DOCX)
