# Supplementary material for: Electro dialysis reversal (EDR) performance for reject brine treatment of reverse osmosis desalination system
Source: PLoS One. 2022 Aug 24;17(8):e0273240. doi: 10.1371/journal.pone.0273240 (PMC9401187; doi:10.1371/journal.pone.0273240)
Supplement: S1 Table — (DOCX) [file pone.0273240.s001.docx]

**Supporting Information File**

**for**

**Electro Dialysis Reversal (EDR) Performance for Reject Brine Treatment of Reverse Osmosis Desalination System**

Hossein Ataei Far^a^, Amir Hessam Hassani^a^, Lobat Taghavi ^a, *^, Mojtaba Fazeli^b^, Abdollah Rashidi Mehrabadi^b^

^a^ Department of Environmental Science, Faculty of Natural Resources and Environment, Science and Research Branch, Islamic Azad University, Tehran, Iran, [ata96025@gmail.com](mailto:ata96025@gmail.com), ahhassani@srbiau.ac.ir, taghavi_lobat@yahoo.com

^b^ Department of Water and Wastewater Engineering, Faculty of Civil, Water and Environmental Engineering, Shahid Beheshti University, Tehran, Iran, [Fazeli@pwut.ac.ir](mailto:Fazeli@pwut.ac.ir), [A_Rashidi@sbu.ac.ir](mailto:A_Rashidi@sbu.ac.ir)

^*^ Corresponding author: Lobat Taghavi* ([taghavi_lobat@yahoo.com](mailto:taghavi_lobat@yahoo.com); [l.taghavi@srbiau.ac.ir](mailto:l.taghavi@srbiau.ac.ir))

**Table S1- Average of chemical parameters in EDR Bench-Scale**

**(Feed flow rate = 12 L/h, Applied voltage = 24 V,
Water recovery ratio for TDS of Feed 29341 mg/L and Temperature of Feed 26.48°C = 23%)**

| **NO.** | **Parameters** | **Feed Brine** | **Desalinated Water** | **Concentrate** |
| --- | --- | --- | --- | --- |
| 1 | Feed Temperature (°C) | 26.48 | 26.42 | 26.05 |
| 2 | pH | 6.83 | 3.46 | 8.57 |
| 3 | Turbidity NTU | 5.19 | 0.94 | 186 |
| 4 | Electrical conductivity µmhos/cm | 45140 | 2640 | 77480 |
| 5 | Total dissolved solids mg/L at 180 | 29341 | 1716 | 50362 |
| 6 | Total Alkalinity mg/L CaCO3 | 110.4 | 0.008 | 192 |
| 7 | Total hardness mg/L | 3194 | 142 | 6076 |
| 8 | Calcium (Ca) mg/L | 1015.2 | 44.48 | 2017.6 |
| 9 | Magnesium (Mg) mg/L | 159.54 | 7.49 | 250.98 |
| 10 | Nitrate mg/L | 33.25 | 2.29 | 95 |
| 11 | Chloride (Cl) mg/L | 18460 | 710 | 37275 |
| 12 | Sulfate(SO_4_) mg/L | 1500 | 80 | 2750 |

**Table S2- Average of chemical parameters in EDR Bench-Scale**

**(Feed flow rate = 12 L/h, Applied voltage = 24 V,
Water recovery ratio for TDS of Feed 9434 mg/L and Temperature of Feed 13.95°C = 52%)**

| **NO.** | **Parameters** | **Feed water** | **Desalinated Water** | **Concentrate** |
| --- | --- | --- | --- | --- |
| 1 | Water Temperature (°C) | 13.95 | 13.54 | 13.32 |
| 2 | pH | 6.52 | 5.44 | 7 |
| 3 | Turbidity NTU | 0.68 | 2.22 | 2.75 |
| 4 | Electrical conductivity µmhos/cm | 14500 | 2795 | 23550 |
| 5 | Total dissolved solids mg/L at 180 | 9434 | 1817 | 15308 |
| 6 | Total Alkalinity mg/L CaCO3 | 105.4 | 0.008 | 108.2 |
| 7 | Total hardness mg/L (CaCO3) | 3280 | 524 | 5040 |
| 8 | Calcium (Ca) mg/L | 1040 | 165.6 | 1632 |
| 9 | Magnesium (Mg) mg/L | 165.4 | 26.73 | 233.28 |
| 10 | Nitrate mg/L | 53 | 7.45 | 83 |
| 11 | Chloride (Cl) mg/L | 4402 | 624.8 | 7952 |
| 12 | Sulfate(SO_4_) mg/L | 1475 | 300 | 1925 |

**Table S3- Average of chemical parameters in EDR Bench-Scale**

**(Feed flow rate = 12 L/h, Applied voltage = 6 V,
Water recovery ratio for TDS of Feed 6764 mg/L and Temperature of Feed 12.91°C = 75%)**

| **NO.** | **Parameters** | **Feed water** | **Desalinated Water** | **Concentrate** |
| --- | --- | --- | --- | --- |
| 1 | Water Temperature (°C) | 12.91 | 12.4 | 12.6 |
| 2 | pH | 8.3 | 3.3 | 2 |
| 3 | Electrical conductivity µmhos/cm | 10410 | 4254 | 21590 |
| 4 | Total dissolved solids mg/L at 180 | 6764 | 2765 | 14042 |
| 5 | Total Alkalinity mg/L CaCO3 | 108 | 0 | 0 |
| 6 | Total hardness mg/L (CaCO3) | 1430 | 436 | 2582 |
| 7 | Calcium (Ca) mg/L | 488 | 134.7 | 857.6 |
| 8 | Magnesium (Mg) mg/L | 51.07 | 24.13 | 106.5 |
| 9 | Sodium (Na) mg/L | 1813 | 675 | 2825 |
| 10 | Potassium (K) mg/L | 5 | 2.5 | 10 |
| 11 | Sulfate(SO_4_) mg/L | 550 | 200 | 950 |

**Table S4- Average of chemical parameters in EDR Bench-Scale**

**(Feed flow rate = 12 L/h, Applied voltage = 12 V,
Water recovery ratio for TDS of Feed 6764 mg/L and Temperature of Feed 12.91°C = 75%)**

| **NO.** | **Parameters** | **Feed water** | **Desalinated Water** | **Concentrate** |
| --- | --- | --- | --- | --- |
| 1 | Water Temperature (°C) | 12.91 | 12.50 | 12.9 |
| 2 | pH | 8.3 | 2.7 | 2 |
| 3 | Electrical conductivity µmhos/cm | 10410 | 1765 | 24040 |
| 4 | Total dissolved solids mg/L at 180 | 6764 | 1147 | 15624 |
| 5 | Total Alkalinity mg/L CaCO3 | 108 | 0 | 0 |
| 6 | Total hardness mg/L (CaCO3) | 1430 | 94 | 2904 |
| 7 | Calcium (Ca) mg/L | 488 | 32.16 | 944 |
| 8 | Magnesium (Mg) mg/L | 51.07 | 3.31 | 132.3 |
| 9 | Sodium (Na) mg/L | 1813 | 125 | 3500 |
| 10 | Potassium (K) mg/L | 5 | 5.2 | 10 |
| 11 | Sulfate(SO_4_) mg/L | 550 | 45 | 1100 |

**Table S5- Average of chemical parameters in EDR Bench-Scale**

**(Feed flow rate = 12 L/h, Applied voltage = 18 V,
Water recovery ratio for TDS of Feed 6764 mg/L and Temperature of Feed 12.91°C = 75%)**

| **NO.** | **Parameters** | **Feed water** | **Desalinated Water** | **Concentrate** |
| --- | --- | --- | --- | --- |
| 1 | Water Temperature (°C) | 12.91 | 12.7 | 12.5 |
| 2 | pH | 8.3 | 3.1 | 2.1 |
| 3 | Electrical conductivity µmhos/cm | 10410 | 653.8 | 23340 |
| 4 | Total dissolved solids mg/L at 180 | 6764 | 425 | 15172 |
| 5 | Total Alkalinity mg/L CaCO3 | 108 | 0 | 0 |
| 6 | Total hardness mg/L (CaCO3) | 1430 | 28 | 2940 |
| 7 | Calcium (Ca) mg/L | 488 | 9/6 | 944 |
| 8 | Magnesium (Mg) mg/L | 51.07 | 0.97 | 141.1 |
| 9 | Sodium (Na) mg/L | 1813 | 26 | 3500 |
| 10 | Potassium (K) mg/L | 5 | 0.5 | 10 |
| 11 | Chloride (Cl) mg/L | - | - | - |
| 12 | Sulfate(SO_4_) mg/L | 550 | 20 | 1200 |

**Table S6- Average of chemical parameters in EDR Bench-Scale**

**(Feed flow rate = 12 L/h, Applied voltage = 24 V,
Water recovery ratio for TDS of Feed 6764 mg/L and Temperature of Feed 12.91°C = 75%)**

| **NO.** | **Parameters** | **Feed water** | **Permeate** | **Brine** |
| --- | --- | --- | --- | --- |
| 1 | Water Temperature (°C) | 12.99 | 12,8 | 12.5 |
| 2 | pH | 8.3 | 2.8 | 2.1 |
| 3 | Electrical conductivity µmhos/cm | 10410 | 2324 | 23820 |
| 4 | Total dissolved solids mg/L at 180 | 6764 | 1510 | 15292 |
| 5 | Total Alkalinity mg/L CaCO3 | 108 | 0 | 0 |
| 6 | Total hardness mg/L (CaCO3) | 1430 | 199.2 | 3070 |
| 7 | Calcium (Ca) mg/L | 488 | 64.8 | 1008 |
| 8 | Magnesium (Mg) mg/L | 51.07 | 9.1 | 133.8 |
| 9 | Sodium (Na) mg/L | 1813 | 220 | 3550 |
| 10 | Potassium (K) mg/L | 5 | 0.8 | 12.5 |
| 11 | Chloride (Cl) mg/L | - | - | - |
| 12 | Sulfate(SO_4_) mg/L | 550 | 115 | 1150 |
