# Supplementary material for: Electro dialysis reversal (EDR) performance for reject brine treatment of reverse osmosis desalination system
Source: PLoS One. 2022 Aug 24;17(8):e0273240. doi: 10.1371/journal.pone.0273240 (PMC9401187; doi:10.1371/journal.pone.0273240)
Supplement: S1 Text — (DOCX) [file pone.0273240.s004.docx]

**Electro Dialysis Reversal (EDR) Performance for Reject Brine Treatment of Reverse Osmosis Desalination System**

Hossein Ataei Far^a^, Amir Hessam Hassani^a^, Lobat Taghavi ^a, *^, Mojtaba Fazeli^b^, Abdollah Rashidi Mehrabadi^b^

^a^ Department of Environmental Science, Faculty of Natural Resources and Environment, Science and Research Branch, Islamic Azad University, Tehran, Iran, [ata96025@gmail.com](mailto:ata96025@gmail.com), ahhassani@srbiau.ac.ir, taghavi_lobat@yahoo.com

**Calculation S&DSI for Feed Brine in EDR Bench-Scale**

**The quality parameters of brine feed:**

**Feed Temperature =26.48 (°C)**

**pH=6.8**

**Total dissolved solids= 29341 mg/L**

**Ca^2+^ =1015.2 mg/L**

**Mg^2+^ =159.5 mg/L**

**SO_4_^2-^ =1500 mg/L**

**Cl^1-^ =18460 mg/L**

**Total Alkalinity mg/L Caco3 = 110.2 mg/L**

**The analysis of feed brine and concentrated quality parameters (EDR Bench-Scale) presented as S1 Table.**

**The Stiff and Davis Stability Index of the feed brine (S&DSIc) is calculated as follows:**

**S&DSI_c_ = pH_c_ − pH_s_ Eq. 1**

**Calculation of the concentrate stream’s pH (pHc) using the ratio of alkalinity to free CO2 in the concentrate.**

**Calculation of the pH at which the concentrate stream is saturated with CaCO3 (pHs):**

**pHs = pCa + pAlk+"K" Eq. 2**

**"K" as a function of concentrate ionic strength and feed temperature.**

**Calculation of the Stiff and Davis Stability Index**

**I_f_ =0.5 ∑ m_i_ _*_ z_i_ Eq. 3**

**m_i_ = molal concentration of ion i (mol/kg)**

**z_i_ = ionic charge of ion i**

**The conversion is as follows:**

**m_i_ = c_i_ ÷ (1,000 MW_i_ ) Eq. 4**

**c_i_ = concentration of ion i in mg/L**

**MW_i_ = molecular weight of ion i**

**Steps to calculate the Stiff and Davis Stability Index of the brine feed-in
 EDR bench-scale**

**Step 1: Calculate the ionic strength I_f_ of the feed brine**

**I_f_ =0.5 ∑ m_i_ _*_ z_i_**

**I_f_ =0.623**

**Step 2: Determine the "K” for the Stiff and Davis Stability Index (S&DSI_c_) :**

**S&DSI_c_ = pH_c_ − pH_s_**

**pHs = pCa + pAlk+"K"**

**With the ionic strength (I_f_) and temperature of feed brine by S&DSI graph
, determine of "K", accordingly:**

**K=3.25**

**Step 3: Determine the pH values for the Stiff and Davis Stability Index (S&DSI_c_) :**

**pCa =1.6**

**pAlk=2.68**

**pH_s_ = pCa + pAlk+"K"**

**pH_s_ =1.6+2.68+3.25**

**pH_s_ =7.53**

**Step 4: Determine the Stiff and Davis Stability Index (S&DSI_c_) :**

**S&DSI_c_ = pH_c_ − pH_s_**

**S&DSI_c_ = 6.8 − 7.53=-0.73**

**Result: The feed brine does not have the scaling potential for calcium carbonate in EDR Bench-scale**
